# Supplementary material for: Coordinated host-pathogen transcriptional dynamics revealed using sorted subpopulations and single macrophages infected with Candida albicans
Source: Nat Commun. 2019 Apr 8;10:1607. doi: 10.1038/s41467-019-09599-8 (PMC6453965; doi:10.1038/s41467-019-09599-8)
Supplement: Supplementary file 3 — Description of Additional Supplementary Files [file 41467_2019_9599_MOESM3_ESM.pdf]

Description of Additional Supplementary Files

File Name: Supplementary Data 1
Description: Sequencing results summary for sorted subpopulation and single-cells

File Name: Supplementary Data 2
Description: Differentially expressed genes in subpopulations of Candida albicans during interaction with macrophages

File Name: Supplementary Data 3
Description: Gene Ontology (GO) enrichment analysis of genes differentially expressed in subpopulations of Candida albicans during interaction with macrophages

File Name: Supplementary Data 4
Description: Differentially expressed genes in subpopulations of macrophages infected with Candida albicans

File Name: Supplementary Data 5
Description: Functional biological categories enriched in macrophages exposed or infected with Candida albicans

File Name: Supplementary Data 6
Description: Genes differentially expressed in single macrophages infected with Candida albicans

File Name: Supplementary Data 7
Description: Genes differentially expressed in Candida albicans phagocytosed by single macrophages

File Name: Supplementary Data 8
Description: Gene ontology (GO) enrichment analysis of genes differentially expressed in phagocytosed Candida albicans in single macrophages

File Name: Supplementary Data 9
Description: Gene expression patterns in single infected macrophages

File Name: Supplementary Data 10
Description: Differential splicing in single infected macrophages

File Name: Supplementary Data 11
Description: Genes expression patterns in single macrophage-phagocytosed Candida albicans
