# Supplementary material for: Coordinated host-pathogen transcriptional dynamics revealed using sorted subpopulations and single macrophages infected with Candida albicans
Source: Nat Commun. 2019 Apr 8;10:1607. doi: 10.1038/s41467-019-09599-8 (PMC6453965; doi:10.1038/s41467-019-09599-8)
Supplement: Supplementary file 15 — Reporting Summary [file 41467_2019_9599_MOESM15_ESM.pdf]

nature research

Corresponding author(s): Christina Cuomo, Reeta Rao

Reporting Summary

Nature Research wishes to improve the reproducibility of the work that we publish. This form provides structure for consistency and transparency in reporting. For further information on Nature Research policies, see Authors & Referees and the Editorial Policy Checklist.

Statistical parameters

When statistical analyses are reported, confirm that the following items are present in the relevant location (e.g. figure legend, table legend, main text, or Methods section).

n/a	Confirmed	
☐	☒	The exact sample size (*n*) for each experimental group/condition, given as a discrete number and unit of measurement
☐	☒	An indication of whether measurements were taken from distinct samples or whether the same sample was measured repeatedly
☐	☒	The statistical test(s) used AND whether they are one- or two-sided<br>*Only common tests should be described solely by name; describe more complex techniques in the Methods section.*
☒	☐	A description of all covariates tested
☐	☒	A description of any assumptions or corrections, such as tests of normality and adjustment for multiple comparisons
☐	☒	A full description of the statistics including central tendency (e.g. means) or other basic estimates (e.g. regression coefficient) AND variation (e.g. standard deviation) or associated estimates of uncertainty (e.g. confidence intervals)
☐	☒	For null hypothesis testing, the test statistic (e.g. *F*, *t*, *r*) with confidence intervals, effect sizes, degrees of freedom and *P* value noted<br>*Give P values as exact values whenever suitable.*
☒	☐	For Bayesian analysis, information on the choice of priors and Markov chain Monte Carlo settings
☒	☐	For hierarchical and complex designs, identification of the appropriate level for tests and full reporting of outcomes
☐	☒	Estimates of effect sizes (e.g. Cohen's *d*, Pearson's *r*), indicating how they were calculated
☒	☐	Clearly defined error bars<br>*State explicitly what error bars represent (e.g. SD, SE, CI)*

Our web collection on statistics for biologists may be useful.

Software and code

Policy information about availability of computer code

Data collection	Primary, bone derived macrophages (BMDMs) were derived from bone marrow cells collected from the femur and tibia of C57BL/6, female mice, grown in “C10” media as previously described 45 and supplemented with macrophage colony stimulating factor (M-CSF) (ThermoFisher Scientific) at final concentration of 10 ng/ml, to promote differentiation into macrophages. Cultures were then stained with F4/80 (Biolegend) to ensure that ~95% of the culture had differentiated into macrophages. BMDMs were seeded in 6 well plates (Falcon). Two days prior the infection experiment, yeast strains were revived on rich media plates. One day prior to the infection experiment, yeast were grown in 3 ml overnight cultures in rich media at 30 °C. On the day of the infection experiment, macrophages were stained with CellMask Deep Red plasma membrane stain (diluted 1:1000) (ThermoFisher Scientific). Macrophages and stain were incubated at 37 °C for 10 minutes, then macrophages were washed twice in 1X PBS. 2 hours prior to infection, yeast cells were acclimated to RPMI media (no phenol red, ThermoFisher Scientific) at 37 °C prior to the infection. Yeast cells were then counted and seeded in a ratio of 1 C. albicans cell to 2 macrophage cells in C10 media. Yeast and macrophages were then co-incubated at 37 °C (5% C02). At the indicated time point, media was removed via aspiration, 1 ml of 1X TrypLE was added to each well and incubated for 10 minutes. After vigorous manual pipetting, 2 wells for each time point were combined into one tube. Each time point was run in biological triplicate. Samples were then spun down at 37 °C, 300g for 10 minutes and resuspended in 1 ml PBS + 2% FCS and placed on ice until FACS. Unexposed controls for both species were collected as described above, not sorted and resuspended in 600 ul of buffer RLT (Qiagen) + 1% β-Mercaptoethanol (Sigma).<br><br>Fluorescence-activated cell sorting (FACS): Samples were sorted on the BDSORP FACSAria into 1X PBS and then frozen at -80 until RNA extraction. Single cells were sorted into 5 ul of RLT 1% β-Mercaptoethanol in a 96 well plate (Eppendorf) and frozen at -80.

cDNA synthesis and library generation: For population samples, the RT reaction was carried out with the following program, as described46, with the addition of RNAse inhibitor (ThermoFisher) at 40U/ul. cDNA was generated from single cells based on the Smart-seq2 method as described previously 47, with the addition of RNase inhibitor was used at 40 U/ul (ThermoFisher) and 3.4 ul of 1 M trehalose was added to the RT reaction. All libraries were constructed using the Nextera XT DNA Sample Kit (Illumina) with custom indexed primers as described 47. Infection subpopulation samples were sequenced on an Illumina Nextseq (37 x 38 cycles). Candida only samples were sequenced on an Illumina Miseq (75 x 75 cycles). Single infected cells were sequenced on Illumina's Nextseq (75x75 cycles).

Data analysis

Read processing and transcript quantification: Picard v.1.107, Bcl2Fastq v.2.17.1.14, BWA v.0.7.10-r789, Bowtie2 v.2.2.1, RSEM v.1.2.21

Differential gene expression analysis: edgeR v.3.10.5 Trinity v.2.1.1, R Studio v.1.0.143, Seurat v.2, scDD v.1.2.0, Monocle v2.8, BRIE v0.2

Functional analysis: Candida Genome Database http://www.candidagenome.org, Ingenuity Pathway analysis (IPA) v.43605602

For manuscripts utilizing custom algorithms or software that are central to the research but not yet described in published literature, software must be made available to editors/reviewers upon request. We strongly encourage code deposition in a community repository (e.g. GitHub). See the Nature Research guidelines for submitting code & software for further information.

Data

Policy information about availability of data

All manuscripts must include a data availability statement. This statement should provide the following information, where applicable:

- Accession codes, unique identifiers, or web links for publicly available datasets
- A list of figures that have associated raw data
- A description of any restrictions on data availability

All sequence data for this project has been deposited in the SRA under Bioproject PRJNA437988.
Raw and processed data for gene expression analysis was deposited in the GEO under GSE111731.
The CAI4-F2-Neut5L-NAT1-mCherry-GFP reporter strain is available from the ATCC Biodefense and Emerging Infections Research Resources Repository (NR-51634), from the Fungal Genetics Stock Center (FGSC#26694).

Field-specific reporting

Please select the best fit for your research. If you are not sure, read the appropriate sections before making your selection.

☒ Life sciences ☐ Behavioural & social sciences ☐ Ecological, evolutionary & environmental sciences

For a reference copy of the document with all sections, see nature.com/authors/policies/ReportingSummary-flat.pdf

Life sciences study design

All studies must disclose on these points even when the disclosure is negative.

Sample size	44 mixed host-pathogen samples for RNA-Seq analysis and 251 mixed host-pathogen samples for single-cell RNA-Seq analysis
Data exclusions	Samples from subpopulations of dead Candida albicans were excluded in some specific analysis as indicated
Replication	For RNA-Seq analysis we performed three biological replicates for each of the infection outcomes
Randomization	Samples were grouped into four distinct infection subpopulations: (i) macrophages infected with live C. albicans (GFP+, mCherry+, Deep red+), (ii) macrophages infected with dead C. albicans (GFP-, mCherry+, Deep red+), (iii) macrophages exposed to C. albicans (GFP-, mCherry-, Deep red+) and (iv) C. albicans exposed to macrophages (GFP+, mCherry+, Deep red-)
Blinding	We were blinded during data collection and analysis

Reporting for specific materials, systems and methods

Materials & experimental systems

n/a	Involved in the study
☐	☒ Unique biological materials
☒	☐ Antibodies
☒	☐ Eukaryotic cell lines
☒	☐ Palaeontology
☒	☐ Animals and other organisms
☒	☐ Human research participants

Methods

n/a	Involved in the study
☒	☐ ChIP-seq
☐	☒ Flow cytometry
☒	☐ MRI-based neuroimaging

Unique biological materials

Policy information about availability of materials

Obtaining unique materials

All the strains sequenced and analyzed in this study are available at the Broad Institute of MIT and Harvard. In addition, all sequence data for this project has been deposited in the SRA under Bioproject PRJNA437988.
Raw and processed data for gene expression analysis was deposited in the GEO under GSE111731.
The CAI4-F2-Neut5L-NAT1-mCherry-GFP reporter strain is available from the ATCC Biodefense and Emerging Infections Research Resources Repository (NR-51634), from the Fungal Genetics Stock Center (FGSC#26694).

Flow Cytometry

Plots

Confirm that:

☐ The axis labels state the marker and fluorochrome used (e.g. CD4-FITC).

☐ The axis scales are clearly visible. Include numbers along axes only for bottom left plot of group (a 'group' is an analysis of identical markers).

☐ All plots are contour plots with outliers or pseudocolor plots.

☐ A numerical value for number of cells or percentage (with statistics) is provided.

Methodology

Sample preparation

Samples were sorted on the BDSORP FACSAria into 1X PBS and then frozen at -80 until RNA extraction. Single cells were sorted into 5 ul of RLT 1% β-Mercaptoethanol in a 96 well plate (Eppendorf) and frozen at -80.

Instrument

BDSORP FACSAria

Software

BD FACSDIVA8.0

Cell population abundance

Cell population abundance varied for each sorted infected subpopulation and time point.

Gating strategy

(i) macrophages infected with live C. albicans (GFP+, mCherry+, Deep red+), (ii) macrophages infected with dead C. albicans (GFP-, mCherry+, Deep red+), (iii) macrophages exposed to C. albicans (GFP-, mCherry-, Deep red+) and (iv) C. albicans exposed to macrophages (GFP+, mCherry+, Deep red-)

☒ Tick this box to confirm that a figure exemplifying the gating strategy is provided in the Supplementary Information.
